# Regadenoson-Stress Dynamic Myocardial Perfusion Improves Diagnostic Performance of CT Angiography in Assessment of Intermediate Coronary Artery Stenosis in Asymptomatic Patients

**DOI:** 10.1155/2015/105629

**Published:** 2015-07-05

**Authors:** Jan Baxa, Milan Hromádka, Jakub Šedivý, Lucie Štěpánková, Jiří Moláček, Bernhard Schmidt, Thomas Flohr, Jiří Ferda

**Affiliations:** ^1^Department of Imaging Methods, University Hospital Pilsen, Alej Svobody 80, 304 60 Pilsen, Czech Republic; ^2^Department of Cardiology, University Hospital Pilsen, Alej Svobody 80, 304 60 Pilsen, Czech Republic; ^3^Department of Internal Medicine, University Hospital Pilsen, Alej Svobody 80, 304 60 Pilsen, Czech Republic; ^4^Department of Surgery, University Hospital Pilsen, Alej Svobody 80, 304 60 Pilsen, Czech Republic; ^5^Siemens Healthcare, CT Physics and Applications Development, Siemensstrasse 1, 91301 Forchheim, Germany

## Abstract

The prospective study included 54 asymptomatic high-risk patients who underwent coronary CT angiography (CTA) and regadenoson-induced stress CT perfusion (rsCTP). Diagnostic accuracy of significant stenosis (≥50%) determination was evaluated for CTA alone and CTA + rsCTP in 27 patients referred to ICA due to the positive rsCTP findings. Combined evaluation of CTA + rsCTP had higher diagnostic accuracy over CTA alone (per-segment: specificity 96 versus 68%, *p* = 0.002; per-vessel: specificity 95 versus 75%, *p* = 0.012) and high overruling rate of rsCTP was proved in intermediate stenosis (40–70%). Results demonstrate a significant additional value of rsCTP in the assessment of intermediate coronary artery stenosis found with CTA.

## 1. Introduction

Computed tomography is routinely used for the detection of coronary heart disease (CHD) with an excellent prognostic value [1]. CT angiography (CTA) of the coronary arteries achieves high quality in the detection of significant stenosis in comparison with invasive coronary angiography (ICA) as the reference method [2]. In several multicentric studies a high prognostic value was also demonstrated in patients with suspected or known CHD [3, 4]. However, because of the small diameter of the coronary arteries, the precise quantification of stenosis is still very difficult, particularly if the quality of the examination is not optimal or the level of noise is higher. Furthermore, the evaluation in coronary artery diameters is also operator dependent and related to the experience. Although the quality of imaging is still increasing, the assessment of stenosis caused by heavily calcified plaques is always really challenging.

Recently, the stress CT perfusion (CTP) examination has become a fast-developing method for the functional assessment of an occlusive CHD. A number of studies have already shown a high image quality and diagnostic performance of stress CTP in the assessment of myocardial perfusion in patients with known or clinically suspected CHD [5].

The aim of our study was to assess the contribution of a combined protocol including CTA and regadenoson-induced stress CT perfusion (rsCTP) to diagnostic performance of significant coronary artery stenosis detection in comparison to CTA evaluation.

## 2. Material and Methods

### 2.1. Patients and Study Design

A prospectively conducted study included 54 consecutive patients (44 males, 10 females, mean age 63 ± 7 years) at high risk of CHD. Concrete inclusion criteria were (1a) peripheral arterial disease (PAD) in severe stage (Fontaine stages IIb–IV) referred to vascular surgery on aorta and/or iliac arteries or (1b) patients with abdominal aortic aneurysm (AAA) referred to resection, (2) no previous history of CHD or recent clinical symptoms, and (3) patients with sinus rhythm. Patients with contraindication to administration of iodinated contrast media were excluded: previous severe allergic adverse reaction and renal dysfunction (creatinine >120 *μ*mol/L or glomerular filtration rate <60 mL/min/1.73 m^2^). Patients were also screened for contraindications of regadenoson administration: atrioventricular block grades II-III and bronchial asthma.

All patients enrolled in the study underwent combined examination protocol comprising coronary CTA, regadenoson-induced stress CT perfusion (rsCTP) of myocardium, and eventually rest CTP. In a subset of enrolled patients results were compared with ICA that was performed in patients with stress-induced hypoperfusion finding or other indications. The study was approved by the local ethics review board and patients signed informed consent.

### 2.2. CT Scanning Protocol

All examinations were performed on a second-generation dual-source CT scanner (SOMATOM Definition Flash, Siemens Healthcare, Forchheim, Germany) with *z*-axis flying focal spot technique. Premedication of beta-blockers or nitrates was not performed in any patient.

#### 2.2.1. Coronary CT Angiography

CTA was performed using retrospective ECG gating with the following parameters: 2 × 2 × 64 × 0.6 mm detector collimation, 280 ms rotation time, 0.2–0.4 adaptive pitch factor, and automated modulation of tube voltage and tube current using the CARE kV and CARE Dose4D (Siemens Healthcare, Forchheim, Germany) with 320 reference mAs/rotation. Automated tube current modulation during R-R interval was used during acquisition. The bolus timing test was performed with 10 mL of contrast medium bolus. The time attenuation curve was analyzed (Syngo DynEva, Siemens Healthcare, Forchheim, Germany) for the adjustment of optimal scanning start (4 sec were added to the time to maximal attenuation). Iodine contrast medium of 50 mL (iomeprol, 400 mgI/mL, Bracco, Milan, Italy) was injected at a rate of 6 mL/sec with 50 mL saline flush at the same rate for CTA scan. Image series of 0.75 mm section width, reconstruction increment 0.4 mm, and convolution filter for vessels (I20f) were reconstructed using iterative reconstruction algorithm (SAFIRE, Siemens Healthcare, Forchheim, Germany).

#### 2.2.2. Dynamic Acquisition of CTP

In the next phase, dynamic scan using two alternating table positions (“shuttle-mode”) was performed in the maximal coverage (73 mm) of the left ventricle myocardium during stress activity. The dynamic protocol was delayed 60 sec after slow manual intravenous application of 400 *μ*g of regadenoson and consists of 15 repeated scans in 30 sec covering left ventricle myocardium (128 × 0.6 mm collimation, rotation 280 ms, 100 kV, and 350 mAs/rotation). Dynamic acquisition was triggered at 60% of R-R interval with delay (4 sec before time to maximal attenuation in ascending aorta) after the bolus of 35 mL iodinated contrast medium injected at 4 mL/sec followed by 40 mL saline flush at the same rate. Patients were instructed to carry out minimal shallow breathing during the examination.

Actual blood pressure (BP) was checked immediately after the acquisition and evaluation of rsCTP was performed (one from intended readers). In the case of finding any hypoperfused areas, dynamic acquisition was repeated at least in 15 minutes' interval with the same acquisition and contrast medium administration parameters. Series of 3 mm section width (reconstruction increment 2 mm) and soft tissue convolution filter (B22f) were performed for subsequent evaluation.

#### 2.2.3. Invasive Coronary Angiography

ICA was performed within maximal three days' interval after CT examinations using a standard technique and coronary artery stenosis were quantified in a consensus of two interventional cardiologists (more than 10 years of experience). Severity of stenosis was quantified and divided into <50% (nonsignificant) and ≥50% (significant) according to routine praxis. The measurement of fraction flow analysis was not performed in all stenosis, so it was not analysed.

### 2.3. Image Analysis

CTA and CTPs evaluation was performed independently by two radiologists with specialization in cardiac imaging (8 and 12 years of experience) at the multimodality workstation Syngo MMWP (Siemens Healthcare, Forchheim, Germany). Reconsideration of discordant findings was performed in consensus. Interobserver agreement analysis of stenosis severity assessment was performed.

#### 2.3.1. CTA

Identified atherosclerotic plaques were localized in segments of coronary arteries using 16-segment model according to the American Heart Association [6]. Quantitative assessment of stenosis severity was performed according to recommendation of Society of Cardiac Computed Tomography. Percentage of maximal diameter luminal narrowing was divided into three grades: 10–39%, 40–70%, and ≥70% [7]. Stenosis in interval of 40–70% was accurately quantified and final determination of nonsignificant (<50%) and significant (≥50%) stenosis was performed.

#### 2.3.2. CTP and Stenosis Reclassification

Dynamic scans were evaluated using a dedicated software application Syngo Volume Perfusion Body with preset for myocardium (Siemens Healthcare, Forchheim, Germany) that included automatic motion correction algorithm with possibility of manual adjustment. Colour-coded perfusion maps (myocardial blood flow and myocardial blood volume) and time-invariant reconstructions (temporal maximum intensity projections) were saved for further analysis. In addition, 4D-CT display of first-pass perfusion (multiphase multiplanar reconstructions in short axis, 10 mm section width) with narrow window width and center adjusted by readers was performed to detect hypoperfused areas (in comparison to surrounding tissue). The presence of a myocardial perfusion defect was considered when hypoperfusion persisted for more than 3 cycles (heartbeats). In this way, we tried to reduce the number of artifacts. The 17-segment model for left ventricle myocardium (according to the American Heart Association) was used and perfusion defects were described as stress induced (completely reversible or partially reversible in rest CTP) and fixed.

Using fused CTA images and perfusion maps, stenosis and perfusion defects were assessed with maximum emphasis on the correlation of anatomical relationships and areas of the vascular supply to the myocardium. In the presence of stress-induced defect in the territory of the branch with nonsignificant (<50%) stenosis according to CTA, this was reclassified as a significant stenosis. The stenosis determined as significant (≥50%) was reclassified when normal perfusion was found in corresponding territory. Separate evaluation of CTP was not performed.

#### 2.3.3. Statistical Analysis

Continuous data were presented as mean, standard deviation (SD), or range and categorical variables as percentages. The diagnostic accuracy of CTA and CTA + rsCTP in detection of significant stenosis (50% cut-off) comparing ICA as reference standard was expressed by sensitivity, specificity, positive predictive value (PPV), and negative predictive value (NPV) on a per-segment and per-vessel basis. The improvement of sensitivity and specificity after rsCTP reclassification was assessed using McNemar test and net reclassification improvement index (NRI). The Cohen kappa value was used for interobserver agreement assessment. A *p* value of <0.05 is considered a statistically significant difference. Statistical analysis was performed using MedCalc software (Ostend, Belgium).

## 3. Results

### 3.1. Patient Characteristic and CT Examination

All enrolled males were without symptoms of CHD; only two females (4%) stated previous unique episode of atypical chest pain, which was not further investigated. Baseline cohort characteristic is summarized in Table 1. There was a significant increase in the average heart rate after regadenoson injection (from 67 ± 13/min to 93 ± 14/min) in all patients. Average heart rate during rest CTP was slightly higher than during CTA (74 ± 15/min).

In average there was no significant change of BP after regadenoson application (systolic and diastolic BP decreased in 18 patients) and any serious adverse effects were not observed. Mean effective dose for CTA was 3.6 mSv ± 0.9, for rsCTP 8.9 mSv ± 2.4, and for rest CTP 8.4 ± 2.1 (calculated from dose length product using conversion factor of 0.014). Complete results are summarized in Table 2.

Sufficient quality for analysis of CTP was achieved in all patients; in 25 cases (31%) the manual adjustment of motion correction was necessary. Overall 17 (1.6%) segments were not covered within limited range of rsCTP acquisition. In 15 cases it was only basal anterior segment; basal anterior and midanterior segment were missed in only 2 cases. These segments ineligible for evaluation did not mean serious complication for assessment.

### 3.2. CT Findings and Diagnostic Accuracy

ICA was performed in a total of 27 patients in whom relevant stress-induced perfusion defects were observed (24 completely reversible and 3 partially reversible); no completely fixed perfusion defects were proved (all patients underwent complete CTP protocol). Overall 324 nonstenotic and 122 stenotic segments were recognized on CTA: 50 with mild stenosis (10–39%), 46 with intermediate stenosis (40–70%), and 26 with severe stenosis or complete occlusion (>70%). Stress-induced hypoperfusion in corresponding supply area was observed in 25 (96%) of >70% stenotic segments. On the other hand, no stress-induced perfusion defects were observed in segments corresponding to mild stenosis (10–39%). Altogether 14 mild stenotic segments were excluded from evaluation because of conflict of supply area with severe stenosis; altogether 108 stenotic segments were analyzed.

**Figure 1:**
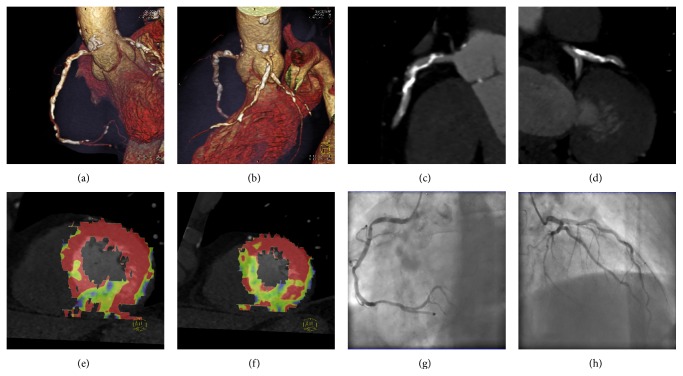
(a–h) 61-year-old male with severe occlusions of iliac arteries was referred to aortobifemoral bypass and without history of CHD symptoms underwent complete CT protocol: coronary CT angiography (CTA), stress CT perfusion (CTP), and rest CTP. Heart rate (HR) during CTA was 71/min (3.1 mSv) and 96/min during stress CTP (5.3 mSv) after 400 micrograms of regadenoson application (60 sec interval). Rest CTP (7.9 mSv) was performed 15 min after stress CTP and HR was 75/min. ICA was performed with 1-day interval. Volume rendering technique images (a, b) show calcified plaques in proximal parts of coronary arteries. Multiplanar reformation images (c, d) show irregular stenosis of the right coronary artery (RCA) and left anterior descending (LAD) artery described as significant using CTA. Colour-coded maps (e, f) of stress myocardial perfusion (blood volume) show perfusion defect in RCA territory and normal perfusion in LAD territory (complete perfusion recovery in rest myocardial perfusion). Invasive coronarography confirmed significant stenosis of RCA (g) and just mild irregularity on LAD (h) artery, CTA decision correctly reclassified by CTP.

Combined evaluation of CTA + rsCTP had higher diagnostic accuracy over CTA alone (per-segment: specificity 96 versus 68%, *p* = 0.002; per-vessel: specificity 95 versus 75%, *p* = 0.012) and high overruling rate of rsCTP was proved in intermediate stenosis (40–70%). During the CTP evaluation, 15 (37%) of 50–70% stenoses were correctly reclassified due to the normal rsCTP as nonsignificant; one stenosis was reclassified falsely (Figure 1). In contrast, the stenoses of 40–49% were correctly reclassified as significant due to the positive rsCTP finding in 2 (40%) cases and falsely in 1 case. Combined evaluation of CTA and rsCTP had higher overall diagnostic accuracy of significant stenosis (50% cut-off). The statistically significant improvement was confirmed in specificity assessment for both per-segment and per-vessel analysis (resp., *p* = 0.002 and *p* = 0.012). The additional value of CTP in severity stenosis reclassification was proved using NRI index, 0.32 (per-segment; *p* < 0.01) and 0.21 (per-vessel; *p* < 0.01), in complete number of stenoses. In a subset of intermediate stenoses (40–69%) the benefit of CTP was higher: 0.66 (per-segment; *p* < 0.01) and 0.68 (per-vessel; *p* < 0.01). Complete results are presented in Tables 3 and 4.

Interobserver agreement regarding stenosis significance assessment was lower in CTA alone assessment (*к* value 0.83) in comparison to combined CTA + CTP assessment (*к* value 0.92).

### 3.3. ICA and Clinical Implications

Altogether 7 patients underwent PCI and 5 patients CABG following results of CT and ICA. In 6 patients PCI and in 6 patients CABG were recommended in case of future development of typical symptoms of angina pectoris.

## 4. Discussion

The assessment of coronary stenosis with moderate severity with a recommendation for further action is the most difficult in the routine practice of CTA. The decision about “functional” significance of stenosis is challenging in particular due to the small diameters of coronary arteries and the presence of image quality worsening factors (motion artefacts and/or calcification). Low specificity and positive predictive value are consequences due to the overestimation of the severity of stenosis caused by atherosclerotic plaques. This fact is the most relevant limitation of CTA, especially with regard to the corresponding risk and exposure of the patient during further examination (e.g., ICA or scintigraphy) in the case of false-positive findings.

The results of our study show the addition of the regadenoson-induced stress perfusion examination to CTA is significantly beneficial for diagnostic performance. An increase of specificity and positive predictive value in the identification of ≥50% stenosis was achieved with the combined evaluation of CTA and rsCTP in comparison to the CTA alone. We did not perform a per-patient analysis (limited number of subjects), but only 2 (8%) patients with a positive CTA + CTP finding were considered as false positive on the basis of the ICA finding. The additional value of CTP assessment is in accordance with most of the previously published papers [8–10]. Moreover, the assumption that the rsCTP will have the greatest benefit in stenosis from 40 to 70% was confirmed.

Kim et al. also demonstrated, in addition to the benefit of stress CTP for diagnostic accuracy, a superior contribution to the detection of ≥50% stenosis, compared with the 70% cut-off value [11]. Although we did not perform the analysis with a >70% cut-off value, the minimal benefit of stress CTP is clear from our results (only one >70% stenosis was overestimated by CTA according to the negative rsCTP). On the other hand, a total of 15 (37%) stenotic segments in the range of 50–70% were correctly reclassified as nonsignificant according to the rsCTP. In previously published studies a different level of diagnostic quality improvement was achieved, but this always involved single-centre studies on patients with varying pretest probability as well as using different designs [12]. The important contribution of our study was a higher rate of borderline and moderately significant stenosis in a cohort (Table 3). This fact increases the importance of the presented results [13].

A limitation of a combined CTA + stress CTP + rest CTP protocol is the higher amount of the administered contrast medium and the radiation dose [14]. The possible methods for reduction of the radiation dose have been widely discussed. A considerable reduction is possible with using single-phase CTP examination, but this technique does not permit the quantification of perfusion parameters, and the quality of defect detection is also questionable. Huber et al. demonstrated high precision in perfusion defect detection when they assessed data sets of single-phase CTP in comparison with dynamic CTP [15]. On the other hand, an experimental study using an animal model demonstrated worse quality in the detection of perfusion defects with a single-phase technique in ≥50% stenosis [16]. Another possibility of significant radiation dose reduction is to skip rest CTP and replace it with CTA that allows the assessment of hypoperfusion and presence of fibrotic scar, which represents chronic (fixed) ischemic lesion. It was not object of research, but our experience is that the CTA could replace rest CTP in the majority of cases. In our study we did not perform rest CTP in cases of completely normal stress CTP, similar to MPI. Using ultralow dose acquisition protocols with a low tube voltage is a very promising procedure; according to the study of Patel et al., radiation exposure could be reduced to 1.9 ± 0.45 mSv, while preserving an adequate image quality [2]. Limited coverage (73 mm) in the *z*-axis has recently been a serious handicap of routine CTP performed on the second-generation dual-source CT scanners. In our cohort, 2% of segments were missed during rsCTP acquisition. Further technical development will certainly eliminate this factor.

A selective a2a blocker (regadenoson) represents a very simple and relatively safe option of pharmacological myocardial stress on a CT workplace, without the necessity of two venous ports, as in adenosine administration. We did not register any further serious undesirable reactions in our patients. In a multicentre study (ADVANCE), a similar effect was demonstrated using a regadenoson bolus injection and an adenosine infusion, which is often used for pharmacological stress in CT or MR [17].

Practical usage of CTP and position in the diagnostic algorithm has not been settled. We chose patients with severe PAD or AAA who are limited for physical exercise. These patients undergo thorough preoperative investigation in our institution considering the high risk of latent CHD including cardiac stress test. The importance of stress test in asymptomatic patients prior to major vascular surgery is a widely discussed topic [18, 19]. Several studies have not confirmed the benefit of prophylactic coronary revascularization and it is not currently recommended to perform a stress test on all patients with negative CHD history before vascular surgery [20, 21]. It is still, however, relevant to consider the preventive performance of a stress test in these patients with a severe involvement of PAD (Fontaine stage IIb and higher), because they are considerably limited in their normal life in terms of physical burden, and so the information about the history of cardiac symptoms has a lower predictive value. Results of our study in particular prevalence of severe CHD are in accordance with the tendency to perform the stress test in this group. In 49 (91%) patients, a minimum of one ≥50% stenosis was found on CTA and stress-induced myocardial hypoperfusion in 27 (50%).

To the best of our knowledge, there is no published study dealing with stress CTP used in a routine diagnostic algorithm replacing another established method. Also, enrolment of high-risk but asymptomatic patients is uncommon. The comparison of stress CTP with established stress tests in cardiology has been performed. Most recent multicentre studies have demonstrated comparable results in detecting the perfusion defects during stress myocardial perfusion imaging with the application of 99mTc-MIBI and stress CTP [22]. Also, in comparison with magnetic resonance, stress CTP results show relatively good diagnostic performance [13, 23]. However, integration of stress CTP in routine diagnostic algorithms in cardiology is still not relevant, but there is the real potential of CT becoming the most complex examination and this fact was approved in our study as well.

Our study has several limitations that have to be mentioned. While detecting perfusion defects, we did not assess the extent within the width of the myocardial wall and no quantification procedure was used [24]. There is no consensus about the appropriate method of quantification assessment. Our evaluation algorithm was established from routine experience, which could be regarded as subjective, but the interobserver agreement in our study was relatively high. Furthermore, the relevance of perfusion quantification in relation to stenosis severity was, until now, assessed in several animal models with promising results [25, 26]. ICA was not performed in all patients, but only on the basis of the CT examination results, which correspond with the routine diagnostic algorithm. Performing ICA in patients with a nonsignificant finding on CTA and above that on rsCTP is not justifiable in the sense of radiation protection. However, we have not included patients without ICA in the evaluation. The ICA analysis did not include FFR (fractional flow reserve) assessment which is considered as the best method for the determination of hemodynamically significant stenosis [27]. A study including the direct comparison of stress CT and stress MPI or MRI should be performed and our group of patients could be suitable, but it was not the goal of our study and also the ICA with FFR procedure is not without risk of complications [28].

## 5. Conclusions

Our results demonstrate a significant additional value of rsCTP in the assessment of intermediate stenosis (40–70%). A combined CTA + rsCTP protocol was also feasible as an alternative stress test with high diagnostic performance in asymptomatic patients at high risk of CHD, referred for major vascular surgery.

## Figures and Tables

**Table 1:** Baseline characteristics.

	All patients (54)	Patients with positive CTP (27)
Age (years)	63 ± 7	62 ± 6
Male	44 (82%)	23 (85%)
PAOD (Fontaine stage)	43 (80%)	24 (89%)
(i) IIb	32	19
(ii) III	9	6
(iii) IV	2	2
AAA	11 (20%)	3 (11%)

Cardiovascular risk factors		
(i) Diabetes	12 (22%)	7 (26%)
(ii) Smoking history	50 (93%)	25 (93%)
(iii) Hyperlipidemia	40 (74%)	19 (70%)
(iv) Hypertension	45 (83%)	21 (78%)
(v) BMI	27.7 ± 4	25.7 ± 6

Age and BMI (body mass index) are mean values ± standard deviation. PAD: peripheral arterial disease; AAA: abdominal aortic aneurysm.

**Table 2:** Heart rate, blood pressure, and radiation exposure.

	CTA/prior regadenoson application (*n* = 54)	rsCTP/post regadenoson application (*n* = 54)	Rest CTP (*n* = 27)
Heart rate (beats/min)	67 ± 13	93 ± 14	74 ± 15
Blood pressure (mm Hg)			
(i) Systolic	143 ± 12	147 ± 20	
(ii) Diastolic	86 ± 9	86 ± 10	
Effective radiation dose (mSv)	3.6 ± 0.9	8.9 ± 2.4	8.4 ± 2.1

Subset group (27) of patients with stress-induced CTP finding who underwent invasive coronary angiography. All parameters are mean values ± standard deviation. CTA: computed tomography angiography; CTP: computed tomography perfusion; rsCTP: regadenoson-induced stress CTP.

**Table 3:** CT and ICA finding in 27 patients.

Segments (108)/vessels (81)	Per-segment analysis				Per-vessel analysis			
	CTA	CTP (stress induced)		ICA	CTA	CTP (stress induced)		ICA
		Positive	Negative	Significant		Positive	Negative	Significant
40–70%	46	29	17	27	23	15	8	13
Nonsignificant (40–49%)	5	3	2	2	3	2	1	2
Significant (50–70%)	41	26	15	25	20	13	7	11
10–39%	36	0	36	0	32	0	32	0
>70%	26	25	1	25	26	25	1	25

≥50%	67	54		52	46	40		38
CTP overruling decision (falsely)		3 (1)	16 (1)			2 (0)	8 (1)	

Only segments with minimal 10% stenosis were included for analysis. Cut-off for significant stenosis was 50%. CTA: computed tomography angiography; CTP: computed tomography perfusion; ICA: invasive coronary angiography.

**Table 4:** Diagnostic accuracy of CTA alone and CTA + CTP to detect significant (≥50%) stenosis.

	Per-segment analysis			Per-vessel analysis		
	CTA	CTA + rsCTP	*p* value	CTA	CTA + rsCTP	*p* value
Sensitivity (%)	96 (48/50)	98 (50/51)	0.625	95 (35/37)	97 (37/38)	0.375
Specificity (%)	68 (39/57)	96 (55/57)	0.002	75 (33/44)	95 (41/43)	0.012
PPV (%)	73 (49/67)	96 (50/52)		76 (35/46)	95 (37/39)	
NPV (%)	95 (39/41)	98 (55/56)		94 (33/35)	98 (41/42)	

McNemar test was used for improvement assessment in sensitivity and specificity. CTA: computed tomography angiography; CTP: computed tomography perfusion.
